# Design Preferences for Mental Health Apps Among Nondigitally Native Adults With Chronic Pain: Qualitative Analysis

**DOI:** 10.2196/87358

**Published:** 2026-08-11

**Authors:** Abby L Cheng, Christine Y Gou, Adriana Martin, Sarah M Hartz, Joanna Abraham

**Affiliations:** 1 Division of Musculoskeletal Physical Medicine and Rehabilitation Department of Orthopaedic Surgery Washington University in St. Louis St. Louis, MO United States; 2 Department of Psychiatry Washington University in St. Louis St. Louis, MO United States; 3 Department of Anesthesiology & Institute for Informatics Washington University in St. Louis St. Louis, MO United States

**Keywords:** anxiety, chronic pain, depression, digital health, digital mental health intervention, engagement, mHealth, middle-age, mobile app, navigation, older adult, usability, user-centered design

## Abstract

**Background:**

On a population level, mental health apps are accessible and effective. However, nondigitally native adults with chronic pain are a large and growing population who have been neglected during the development process of these interventions. Although technology use is rapidly growing among this population, their engagement with mobile health–related apps is lagging because usability is often not optimized for their needs and preferences.

**Objective:**

This study aimed to identify design preferences and determinants of engagement with mental health apps by nondigitally native adults who have chronic pain and coexisting symptoms of depression or anxiety.

**Methods:**

In this qualitative study, participants completed a semistructured interview regarding their experience with, and perceptions of, mobile devices, apps, and digital health interventions. Participants were 45 years or older; scored ≥10 on the 9-item Patient Health Questionnaire, 7-item Generalized Anxiety Disorder, or both; endorsed pain on most days or every day in the past 3 months; and were living in the United States. The interview guide was informed by the Consolidated Framework for Implementation Research and the Behavioral Intervention Technology model. Codes were organized into themes. Recruitment continued until thematic saturation was achieved.

**Results:**

A total of 42 participants were interviewed (mean age 57, SD 8 years; n=32, 76% women). Participants strongly preferred apps that are free, describe strong privacy policies, and add functional value to their lives. They were more motivated by “real-life” goal achievement and tangible health improvements than by gamification within an app, and many were wary of allowing apps to passively collect certain types of data, especially to make inferences about their mental health. Most participants were unaware of apps designed to address chronic pain, but they were interested in the concept, particularly to help track their mood and pain and then identify associations between their symptoms, app activity, and other life events. Despite their daily use of apps, many participants described frequent challenges related to app navigation. While on-demand access to app tools was preferred, most participants appreciated the potential value of occasional push notifications if their timing was thoughtful and customizable. Participants cautioned against an overly cheerful, “infantile,” or informal tone for an app that addresses serious issues such as mental health and chronic pain.

**Conclusions:**

Mental health apps for nondigitally native adults should highlight tangible health improvements that can be achieved from app engagement (more so than gamification), potentially using a multidomain tracking feature, if appropriate. This population is available to receive just-in-time adaptive interventions, but the frequency and timing should be thoughtful, customizable, and not wholly reliant on passively collected personal data. Health-related apps designed to address conditions that are more common with increasing age should account for these preferences.

## Introduction

Chronic pain commonly coexists with depression and anxiety, and the prevalence of chronic pain increases with age. In the United States, nearly one-third of middle-aged and older adults are living with chronic pain, and up to half of them have depression, anxiety, or both [1-4]. Furthermore, chronic pain reduces the effectiveness of stand-alone mental health treatment unless a person’s pain is simultaneously addressed [5,6]. Regardless of the etiology, chronic pain is associated with increased rates of recurrent depressive and anxiety episodes, reduced rates of remission, and reduced efficacy of antidepressant medications [7-10]. For these patients, addressing their mental health and pain experience simultaneously is more effective than addressing either problem in isolation [11].

Additionally, technology use is rapidly growing in these age groups, but engagement with digital health interventions is lagging because usability is often not optimized for their needs and preferences [2,7-10,12-23]. At this time, the majority of middle-aged and older adults are nondigital natives, meaning they were born before 1980 and grew up before immediate access to technology was widespread [24,25]. Compared to younger (and digitally native) users, this population more often has a greater interest in interventions that address multimorbidity; they prefer different screen layouts due to age-related changes in visual acuity, eye tracking patterns, and dexterity (eg, simple and flat navigation structure, large labeled buttons, and minimal need for typing); and they may benefit from more “push” notifications because they check their mobile devices less frequently. Some also have age-related cognitive deficits that reduce their ability to navigate through apps [20,26-31]. Although these age-related preference differences are now well-established, the user design of commercially available digital health apps has often continued to lag behind accepted design recommendations [32].

On a population level, digital mental health interventions, including mobile apps, effectively reduce depression and anxiety symptoms, and they have produced clinical outcomes that are equivalent to face-to-face treatment [17,19,33,34]. They also improve access to mental health support by reducing common barriers due to stigma, transportation, cost, and provider shortages that too often limit access to trained mental health professionals [35,36]. In recent years, the increasing availability of artificial intelligence has only accelerated public interest in using technology to address mental health symptoms [37-41]. Nevertheless, some populations have been neglected during the development process of mental health apps [42], and nondigitally native adults with chronic pain are one such understudied, yet growing and significant population [20].

The purpose of this study was to narrow this evidence gap by exploring design preferences of nondigitally native adults who have chronic pain and coexisting symptoms of depression or anxiety, with particular emphasis on examining determinants of engagement with mental health apps. This study is the first phase of a 3-phase project using the Discover, Design/Build, and Test (DDBT) framework to design and launch a mental health app that is optimized for the usability needs and design preferences of this population [43,44].

## Methods

### Study Design

In this formative qualitative study, each participant completed a single semistructured interview regarding their first-hand experience with, and perceptions of, mobile devices, apps, and digital health interventions [45,46]. Participant interviews occurred between April 3, 2024, and October 2, 2024. Data analysis occurred in 2024 and 2025. The study details are described in accordance with the Consolidated Criteria for Reporting Qualitative Research (COREQ) guidelines [47]. Of note, study participants also completed usability testing of a mental health app and provided feedback on specific usability-related features as part of the 3-phase project. These findings are reported separately [48].

### Ethical Considerations

Prior to participant recruitment, approval of the study and all related procedures was obtained from the Washington University Institutional Review Board (IRB; 202311024). Participant consent was obtained via a waiver of written informed consent, and participants’ data were maintained in secure databases and were deidentified before sharing with anyone outside the IRB-approved study team. Participants received a US $30 gift card as remuneration for participation.

### Participants

To be eligible, potential participants had to be at least 45 years of age, report at least moderate depression or anxiety symptoms (9-item Patient Health Questionnaire [PHQ-9] score of ≥10, 7-item Generalized Anxiety Disorder [GAD-7] score of ≥10, or both), and endorse chronic pain (defined as pain on most days or every day in the past 3 months) [2,49,50]. Potential participants were excluded if they endorsed an active mental health crisis that warranted escalation of care (eg, active suicidal ideation and psychosis), had cognitive impairment that would interfere with communication with the interviewer, or lived outside the United States.

### Recruitment

Potential participants were recruited from a variety of sources including from a chronic musculoskeletal pain clinic at an academic medical center (ie, printed flyers in clinic, phone call or email from the research team), email and social media advertisement via a university-based volunteer research participant registry (ie, Facebook and Twitter/X), and targeted advertisement in the electronic newsletter of a free online health community (The Mighty). Initial eligibility screening was performed using a self-reported questionnaire hosted by REDCap (Vanderbilt University), followed by final verification by a study team member [51,52]. Recruitment and interviews continued until thematic saturation had been achieved.

### Guiding Frameworks

Development of the interview guide was informed by the CFIR (Consolidated Framework for Implementation Research) and the Behavioral Intervention Technology (BIT) model [53,54]. The CFIR was used to ensure that all domains that could impact users’ engagement with a mental health app were systematically addressed (ie, characteristics of the app user [individual], the user’s home environment [inner setting], the user’s social and structural support environment [outer setting], the user’s preferred design features of the app [adaptable periphery], the app’s therapeutic content [intervention], and the process of identifying, downloading, and onboarding to a new app [implementation process]) [53]. The BIT model was used to ensure that participants were prompted to comment on how an app’s tools [elements], user design [characteristics], and intended timing of interactions [workflow] may impact their engagement with the app’s behavioral interventions [54].

### Interview Process

Semistructured interviews were conducted and recorded using audio/visual conferencing via a secure Zoom platform, each lasting approximately 45 to 60 minutes. The virtual interview format facilitated more accessible participation for people with mobility limitations related to pain, other medical conditions, transportation barriers, or geographic distance from the study site. Participants were also asked to answer sociodemographic descriptive questions via an electronic REDCap questionnaire, either before or after completing the interview. During the interviews, only the interviewer and participant were present. The interviewer described the rationale and purpose of the study, and then participants were asked questions from the interview guide (Multimedia Appendix 1).

### Analysis

Audio recordings of the interviews were professionally transcribed [55], cleaned, and chunked. A preliminary codebook was developed by the senior investigator using a deductive approach guided by the CFIR and BIT models (ALC). The codebook and coding tree were refined using an inductive approach as 3 research team members coded 5 transcripts together (ALC, AM, and CYG). At that time, >90% consensus was reached, and there were fewer than 5 discrepancies between coders. After these discrepancies were discussed and resolved through consensus, the codebook was refined accordingly, and the rest of the transcripts were each coded by a single team member (AM or CYG) using NVivo 15 (Lumivero). Periodic coding cross-checks were performed across team members to ensure consistency. Next, we used thematic analysis to capture patterns in the data by examining relationships between codes [56]. Codes were organized into themes by a primary and secondary research team member. Participant recruitment, data collection via interviews, and data analysis were iterative and continued until thematic saturation was reached [56]. This was defined as the point at which no new themes or insights emerged from analysis of the last 3 interviews or related transcripts. Finalization of the thematic analysis was performed as a group discussion among 3 research team members (AM, CYG, and ALC), using the BIT model as a framework for organization. To optimally facilitate rapid intervention development and refinement as part of our 3-phase project, we aimed to identify and highlight both overarching themes and specific feature preferences among this population that could be implemented and iteratively tested using the DDBT framework.

### Reflexivity, Rigor, and Reproducibility

The senior investigator was a female physician scientist with a clinical background in physiatry and musculoskeletal medicine (ALC). The interviews were conducted by a senior female research coordinator who has clinical research experience working with older adults and in the field of mental health (AM). The interviewer was not involved in clinical care, and participants’ only contact with the interviewer was related to study recruitment, consent, and participation. The third member of the coding and analysis team was a female physiatry resident trainee (CYG). Additionally, a clinician scientist with mental health clinical expertise (SMH) and a health IT expert (JA) were involved in finalizing the study design, interview guide, and interpretation of themes. Although formal member checking was not possible due to the single-session nature of participant involvement, informal member checking was performed by the interviewer by paraphrasing participants’ statements during interviews to ensure the participants’ thoughts and opinions were correctly understood. Additionally, preliminary themes from early interviews were iteratively shared with later participants, who generally affirmed their face validity.

## Results

### Overview

Of 62 people who completed the screening questionnaire and were determined to be eligible, 42 participants who represented a range of sociodemographic backgrounds were invited and participated in the study, at which point thematic saturation had been achieved (mean age 57, SD 8 years; n=32, 76% women; at least n=28, 67% non-Hispanic White; n=40, 96% used the internet at least multiple times a day; Table 1). We identified themes that cut across the CFIR domains and related to factors that impact participants’ general use of mobile devices and apps, in addition to determinants of engagement with health-related apps and specifically mental health apps.

**Table 1:** Participant characteristics (N=42).

Characteristic			Value
Age (years), median (IQR; range)			55 (51-61; 45-76)
Sex^a^, n (%)			
	Female	32 (76)	
	Male	10 (24)	
Race, n (%)			
	White	30 (71)	
	Black or African American	9 (21)	
	Other	1 (2)	
	More than one race	1 (2)	
	Prefer not to answer	1 (2)	
Ethnicity, n (%)			
	Hispanic or Latino	3 (7)	
	Not Hispanic or Latino	36 (86)	
	Unknown or prefer not to answer	3 (7)	
Employment status, n (%)			
	Disabled	13 (31)	
	Working full-time	11 (26)	
	Retired	10 (24)	
	Working part-time	4 (10)	
	Unemployed	2 (5)	
	Prefer not to answer	2 (5)	
Frequency of internet use^b^, n (%)			
	Most of the day	12 (29)	
	Multiple times a day	28 (67)	
	At least once a day	1 (2)	
	Less than once a day	1 (2)	
Duration of chronic pain (years), median (IQR; range)			12 (8-20; 2-65)
High-impact chronic pain present^c^, n (%)			33 (80)
BPI^d^ Pain Interference score, median (IQR; range)			7.1 (6-8.1; 2.4-9)
PHQ-9^e^ score, median (IQR; range)			14 (12-17; 7-22)
GAD-7^f^ score, median (IQR; range)			12 (9-15; 1-21)
Prior mental health treatments, n (%)			
	Medication	30 (71)	
	Psychiatrist, psychologist, or therapist	28 (67)	
	Self-management (meditation, mindfulness, deep breathing, etc)	21 (50)	
	Support group	10 (24)	
	Mental health app	4 (10)	
	None	3 (7)	

^a^Sex and gender identity were concordant for all participants.

^b^Self-reported internet use was captured by the Pew Digital Savviness Classifier [57].

^c^”High impact chronic pain” is defined as pain that limits life or work activities on most or every day over the past 3 months [2].

^d^The Brief Pain Inventory (BPI) Pain Interference score ranges from 0 to 10 [58]. Higher scores indicate greater pain interference.

^e^The 9-item Patient Health Questionnaire (PHQ-9) is a 9-item screening measure for depressive symptoms [49]. Scores range 0-27. Higher scores suggest more depressive symptoms.

^f^The 7-item Generalized Anxiety Disorder (GAD-7) is a 7-item screening measure for anxiety symptoms [50]. Scores range 0-21. Higher scores suggest more anxiety symptoms.

### General Use of Mobile Devices and Apps

#### Users Are Constantly Connected

Our nondigitally native adult participants reported having their mobile devices within arm’s reach essentially during all waking hours, largely out of necessity for daily life activities such as finances and urgent communication, as well as for leisure especially related to social connection (Table 2). Most participants reported typically responding to mobile device messages immediately or at least within the same day.

**Table 2:** General patterns of mobile device and app use among nondigitally native adults who have chronic pain and coexisting symptoms of depression or anxiety.

Theme		Representative quote
Tools		
	Life navigation: participants typically used apps “because they have to” *for daily life* (eg, for driving directions, banking, taxes, shopping, coupons, alarm clocks, current events, and having a contact method in case of emergencies).	“I pretty much use my phone for absolutely every aspect of my life...work, grocery list, like everything.” (48-year-old male)
	Leisure: participants also frequently used apps that facilitate *social connection and entertainment* (eg, social media, video chatting, taking pictures, shopping, and, less commonly, to play games).	“Keep in touch with other people, movies and TV, sometimes books, sometimes for my grandkids.” (60-year-old female)
User design		
	Usability challenges with mobile devices: participants commonly experienced challenges using their mobile devices due to *dexterity difficulty* with their screen’s size and *forgetting passwords*.	“Remembering my password is definitely [a challenge]. Sometimes finding the right one, because if you put in an AT&T one, sometimes there’s three or four of them and it’s like, ‘Which one is the one that I should actually be downloading?’” (60-year-old female)
	Usability challenges with apps: participants also reported challenges navigating within some apps, understanding *how to take advantage of all an app’s features*, and *reducing unwanted notifications* for some apps.	“Some of the apps are confusing with how they want you to move around, and they don’t really explain it.” (60-year-old female) “Trying to figure out everything that the app does—to make sure I’m using the maximum of everything that it does.” (53-year-old female) “Some of my apps give me notifications that I don’t really want. Figuring out how to turn off unwanted notifications can be a challenge.” (64-year-old female)
	Troubleshooting: when participants have trouble navigating an app, they typically *first “push around” on their own* to troubleshoot. Next, they search the internet for assistance. Finally, they reach out to their social circle or a professional for navigation assistance if needed.	“Pretty much in this order—I will try it on my own. Then I will look up a video because it’s like, ‘I’m determined to do this.’ But then if it’s something too complicated, then usually I call one of my kids.” (53-year-old female)
Timing of interactions		
	Availability for alerts: most participants kept their smartphones *almost always within arm’s reach*, except maybe silenced while sleeping or driving.	“I rarely turn it off.” (60-year-old female)
	Response time: participants typically responded to mobile device messages *immediately or within the same day*.	“[I respond] when I see them, if I need to.” (50-year-old female)
General app preferences		
	Pros: participants preferred apps that are *free, functionally meaningful*, personally relevant, and ideally have good brand reputation.	“I don’t typically pay for a lot of the apps. There’s usually free versions of something out there that you can find...There is a medication that I use that they have you download the app for, and there’s no point to it. There’s no purpose to it, so it’s just taking up space on my phone.” (49-year-old female)
	Cons: participants were less likely to use apps that raise *privacy concerns* about how their data will be used or that frequently display advertisements. Some participants were also less likely to use apps that require high data usage or a stable internet connection.	“Making sure tracking and invasion of privacy is not an issue.” (72-year-old male)

#### Preference for Free, Useful, Trusted Apps

Participants strongly preferred apps that were free. They also gravitated toward apps that were functionally meaningful, personally relevant, and well-established with a good reputation. They reported being less likely to use apps that do not provide sufficient reassurance that users’ privacy and data will be protected. Many also disliked frequent advertisements within apps.

#### Challenges With Navigation and Notifications

Participants reported commonly experiencing usability challenges with mobile devices due to difficulty with dexterity and forgetting passwords. They also described challenges navigating some apps, particularly with regard to understanding how to use all of an app’s features and reducing unwanted notifications from apps. When usability challenges are encountered, participants most frequently reported troubleshooting by “pushing around” on their own, followed by searching the internet for assistance (eg, Google and YouTube), and then reaching out to their social circle or professional assistance as a last resort.

### Determinants of Engagement With Health-Related Apps: Interest in Content and Tracking Capabilities That Result in “Real-Life” Goal Achievement

Participants most frequently reported using health-related apps for education (eg, to learn new information), accountability (eg, for assigned tasks and reminders), and community (eg, to connect with people who are living with similar challenges; Table 3). Tracking capability was a common theme among desired functions of health-related apps, whether to track physical activity, biometrics, mood, food intake, or another health-related measure. Nevertheless, participants insisted on clear communication and ability to choose which types of passive data are collected and for what purposes, due to concerns related to privacy and “creepiness” (eg, acceptable to passively collect step count but not location). Most participants also did not consider gamification with rewards within a health-related app to be a meaningful motivator of behavior change or engagement. However, participants did report being more likely to value and use a health-related app if it is recommended or hosted by a medical professional. Regarding the timing of app interaction, participants highly appreciated the capability to customize the time and frequency of push notifications and other prompts from the app.

**Table 3:** Determinants of engagement with health-related apps among nondigitally native adults who have chronic pain and coexisting symptoms of depression or anxiety.

Theme		Representative quote
Tools		
	Goals: participants used health-related apps for *education, accountability, and community*.	“I primarily enjoy getting information from them.” (61-year-old female) “I’ve done [a fitness app]...and [a mental health app]. I know what I need to do each day, and I don’t have to worry about remembering it. So I can log in, and each day I know I need to tackle these kind of tasks.” (49-year-old female) “I use [an online health community app]. A lot of people have the same experiences I do. So that’s why I like it. The same thing that I go through—people not believing me—it gives you a lot of insights.” (51-year-old female)
	Function: in descending order, participants most commonly used apps for activity/fitness tracking; blood sugar, blood pressure, and weight tracking; mental health, mindfulness, and mood tracking; food/calorie tracking; medication refills and communication with a health care provider; and sleep assistance.	“I have a [fitness tracking] app for monitoring my steps and water. My oncologist, once I was finished with treatment, wanted me to make sure I walked 10 to 15,000 steps a day.” (60-year-old female)
	Tracking: participants predominantly used health-related apps to assist with tracking one or more elements of their health.	“The step counter is really probably the only one I use regularly.” (50-year-old female)
User design		
	Selective enthusiasm for passive data: the majority of participants were *open to passive collection of some types of data but not others* due to privacy concerns. If an app does passively collect data, they strongly voiced a need for the app to clearly describe the purpose and extent upfront, with an option to opt out.	“I tend to steer away from apps that ask me that kind of information because I’m not comfortable being tracked in where I am, what I’m doing. I just find it intrusive. I mean, I put my weight on there, my height, it has my blood pressure and my heartrate—so that’s pretty personal, but location—I do not share... [Passive data tracking] is a little creepy.” (66-year-old female) “I want to be able to pick when I use [passive data]. I don’t want it to be able to use it and pick me. I mean, I need reminders to use [the app], but not by my location.” (62-year-old female)
	Low enthusiasm for gamification: most participants *did not consider embedded games or rewards as a meaningful motivator* to engage with a health-related app.	“I don’t know if I’m a typical guy or maybe not, but I don’t like games. When it comes to something like this, I’m not motivated by a reward. I would rather see [all my tool] options than, ‘Good boy, good boy.’ ...When it comes to counting my steps, it is nice at the end of the night to have hit my target or beyond. So in that sense, yes, an emotional reward is a good thing. But the reward is that I’ve accomplished what I want. I don’t need something extra on top of that.” (72-year-old male)
Timing of interactions		
	Participants strongly preferred the ability to *customize their timing of interaction* with the app (eg, time and frequency of push notifications and ability to turn off push notifications).	“I like if it allows you to set what time you want [for check-ins].” (64-year-old female) “I would like the option [of changing the check in frequency].” (55-year-old female)
General app preferences		
	Source: participants were more likely to use a health-related app if it is *recommended by a medical professional* or directly offered from their medical provider/hospital system.	“It’s normally based on advice from a medical professional that I would download something of this type.” (51-year-old male)

### Determinants of Engagement With Mental Health Apps That Address Chronic Pain

#### Explanation of How “An App Can Help With Pain”

Many participants were unaware of apps that were designed to address chronic pain (in addition to mental health), nor did they understand how an app could address chronic pain (Table 4). When it was explained that such an app could guide users through pain coping strategies, participants were generally enthusiastic and expected that such an app would be helpful. Given the relative unawareness, participants expected that early in the onboarding and orientation process, such an app would provide education on the neuroscience of chronic pain and how it could help them manage and cope with chronic pain. They also expressed interest in tools related to mindfulness, cognitive behavioral strategies to improve pain acceptance, and gentle physical exercise. They generally preferred the app to guide them through a structured curriculum initially, but also to allow for self-guided exploration of app tools, if desired.

**Table 4:** Preferred features of a mental health app that addresses chronic pain among nondigitally native adults.

Theme				Representative quote
Tools				
	Features			
		Participants were interested in having access to a variety of tools including education about chronic pain neuroscience, mindfulness exercises to help manage pain, strategies to improve pain coping and acceptance, pain and mood tracking capabilities, and gentle physical exercise. They appreciated immediate, 24/7 access to management tools.	“I think it’s probably going to be some kind of a help [to have a] mindfulness activity to help control and manage pain.” (56-year-old female) “Give me a summary of how my week was...pain, mood—both depression and anxiety—which I’m sure part of my anxiety is from the pain. Yeah.” (66-year-old female) “Last night I had trouble sleeping because of my lower back, and it’s progressively getting worse. I have injections next week and so on, and I’m really looking forward to them. But in the meantime—getting up in the middle of the night and just saying, ‘My back hurts, maybe there’s this app I can check out.’ Could be interesting.” (51-year-old male)	
	Mood tracking			
		Participants were interested in periodic, brief, self-report assessments to track their mental health over time (eg, 4 questions as opposed to 16 questions). Participants preferred self-reporting their mood, as opposed to the app using passively collected data to infer their mood (eg, with call logs, step count, and GPS data). Participants would be more motivated to complete self-assessments if the results are used to tailor suggestions of additional tools for them. Participants voiced mixed sentiments regarding whether they would want their health care team to see responses to their self-reported mental health assessments.	“If it asked, ‘Hey, how was your week? Did you have any kind of issues that you need to talk about?’ And then if you’re honest with the app, then maybe it can touch base a little bit sooner.” (48-year-old male) “[Passive data collection to infer mood] is a little creepy.” (66-year-old female) “It can be a double-edged sword. For example, if you answer incorrectly, ‘Do you feel like harming yourself?’ Is somebody [from your healthcare team] going to say, ‘Wait a minute, there are specific laws that say we have to take action and you have to be under care now.’ If it’s chronic pain, that’s different. But if it’s mental health, there’s a big stigma with it.” (51-year-old male)	
	Other tracking feature			
		Participants were interested in a feature to track associations between their pain symptoms, mental health symptoms, physical activity and life events, and engagement/tasks completed within the app. Their goal from this complex tracking feature would be to identify what app tools and general behaviors were helpful to reduce their symptoms. Some participants would also want to show this data to their health care team.	“Yeah, maybe my activity level is higher. Maybe my emotional level is better. I would think tracking all of those things that correlate directly to my pain level would be good...Something where you can really see the results of the app working with you or for you would be good, but I think it would feel a little disconnected without also tracking any kind of pain scale.” (56-year-old female)	
	Methods to encourage sustained engagement			
		Participants expected they would be motivated to sustain engagement with the app if they were instructed to set goals and had pending tasks on a to-do list, if the app helped them track their tasks completed and corresponding symptom improvement, and if the app suggested new features for them to try. They were generally not in favor of using access to new features as a reward/motivator for continued engagement. Participants were also in favor of a free trial period of paid content before deciding whether to purchase the app (or an upgrade). There was a slight preference for a content-limited trial rather than a time-limited trial (eg, 2-4 weeks).	“Is there stuff that you could add that you’re working on in the next week, or maybe things that you didn’t fully complete?” (60-year-old male) “Suggest a tool, something that’s actually useful as opposed to a gold star. Yes. Yes.” (53-year-old female) “Yeah. Having a trial and then going to paid content after the trial—that would determine for me, ‘How well is this working?’” (60-year-old female)	
User design				
	Onboarding			
		Participants requested a clear explanation regarding how the app can help with chronic pain and mental health (eg, coping and management strategies, rather than a focus on pain severity reduction).	“I am confused, and I don’t know how this is going to work. Yeah, I guess my knees and my back are preventing me from exercising. Is that what you’re talking about? Mental and physical health going together?” (66-year-old female) “I guess...intervene in letting pain take over. I think by checking in or doing mindfulness things, you could possibly alleviate chronic pain.” (56-year-old female)	
	Tone			
		Some participants thought an overly cheerful, personal tone presented by a cartoon-like avatar was less suitable for older adults and felt artificial. Similarly, participants had mixed opinions on the use of emojis throughout the app experience.	“These kinds of issues, we don’t want to make light of them, and I think a [cartoon-like chatbot avatar] kind of makes it a little childish. If this was geared toward children and teens, ok. But for adults, I think it needs to have something a little more serious... I think I would be more comfortable with more of a question-and-answer screen—more like journal prompts than a text conversation.” (60-year-old female) “For me, [emojis are] very unnecessary. [They’re] infantile. I mean, but I’m old. So a lot of this stuff is goofy now, that wouldn’t be goofy to other people. If a kid is using it, they might like that.” (69-year-old female) “I think an emoji is like putting an expression to a sentence. So I think it’s amazing.” (52-year-old female)	
	User flow			
		Participants generally preferred for the app to initially guide them through a structured curriculum (such as for a duration of 8 weeks) but to also allow for self-guided exploration through the app, if desired.	“It seems like once you become familiar with what it is, self-guided would definitely be preferable.” (56-year-old female) “I think eight weeks would be good because it takes a significant amount of time to change habits or incorporate new habits.” (54-year-old female)	
Timing of interactions				
	Notifications to prompt engagement			
		Most participants preferred to initiate engagement with the app on their own, but many participants were also welcoming or at least accepting of occasional notifications to encourage engagement if relevant. Most participants expressed that once or twice daily notifications would be acceptable, and some participants preferred an even greater frequency of notifications. For once daily notifications, evening check-ins were more often preferred to morning check-ins. Participants voiced varied preferences regarding the option for notification frequency to be adaptive based on their engagement. Some participants were in favor of pausing notifications (eg, for 3-7 days), while others did not want a pause in notifications unless they were specifically asked first.	“If it recognizes that you struggled, and if you’re honest with the app, then maybe it can touch base a little bit sooner.” (48-year-old male) “[Check-ins twice a day] is okay with me because I don’t do anything. I’m in pain, I don’t work, and all I do is go back and forth to the doctors.” (55-year-old female) “Like, you could say, ‘Dismiss for three days,’ or however long it is. Or let’s say you’re going on vacation, you could set a time limit to dismiss for seven days or however long it needs to go. Give me the option to click that button, then I can go from there.” (49-year-old female) With regards to pausing notifications: “Don’t forget about me now. Everybody else in the world does. And don’t give up on me. I look at that as giving up on me. Don’t do that. Don’t leave me.” (62-year-old female)	
General app preferences				
	Improved awareness			
		While most participants were aware of apps designed to address mental health, many participants were unaware of apps that address chronic pain. They were also unclear as to “how an app can help with pain.”	“I had no idea there were any [apps to help manage pain]. I’m wondering what [a pain app] can do because I have no clue.” (61-year-old male)	
	Other stressors to address			
		Participants also suggested additional stressors that should be addressed by an app that aims to improve mental health and chronic pain. These stressors commonly included other health concerns, finances, and low motivation. Mobility limitations, shame, attention issues, frustration, loneliness, time pressures, and work were also suggested.	“It could be other health challenges. You could have something related other than just pain.” (53-year-old female) “Finances play a big part of everything you do because if you don’t have it, you can’t do it.” (55-year-old female)	

#### Interest in Self-Reported Symptom Tracking

Participants were particularly interested in a tracking feature that would help identify associations between their pain, mental health symptoms, daily activities and events, and engagement with the app. To track mood, participants generally preferred completing periodic, brief self-assessments, rather than having the app use passively collected data, such as call logs or step count, to infer their mood, due to concerns about privacy and the accuracy of inferences. Participants reported being motivated to complete these self-assessments if the results lead to a more personalized and relevant app experience. They were somewhat conflicted as to whether they wanted their self-reported symptoms to be shared with their health care team. While many participants anticipated added clinical value from sharing data such as a pain diary, some of these same participants (in addition to others) expressed concern regarding how mental health symptom reports may be handled by licensed health care providers. They feared that a clinician may misinterpret their symptom reports and feel obligated as a mandatory reporter to act on concern for potential imminent danger, even if none exists.

#### Encouraging But Mature Tone

Several participants cautioned against an overly personal or cheerful tone for an app that addresses chronic pain and mental health because it felt artificial and less suitable for their age group. Similarly, participants had mixed opinions on the use of a cartoon-like avatar or emojis throughout the app experience. Some felt they were “infantile,” whereas others felt they enhanced communication. To maximize the relevance of an app that addresses chronic pain and mental health, participants suggested additional stressors that should also be acknowledged and ideally addressed, such as other health issues, financial pressures, and low motivation.

#### Customized Frequency of Notifications

Most participants preferred to initiate engagement with the app on their own, but many participants were also welcoming, or at least accepting, of notifications to encourage engagement if relevant. Participants typically described once- or twice-daily notifications as being most appropriate (especially evening notifications). Most participants were also open to an adaptive frequency of notifications based on their degree of engagement. However, some participants voiced concern about feeling like the app “ghosted [them] just like people do” if notifications suddenly stopped without their explicit request.

#### Motivated by Real-World Results

Regarding sustained engagement with an app that addresses chronic pain and mental health, participants expected to be motivated to continue engaging if they had specific goals and a to-do list within the app, especially if they could see a correlation between task completion within the app and symptom improvement. Participants were not in favor of using access to new app tools as a reward for engagement because they wanted to have access to whichever tool they thought was most relevant at the time they needed it. Instead, participants were generally open to a free trial period of app content, which may then motivate them to purchase access to additional content (or continued access to the original content).

## Discussion

### Principal Results

This qualitative study investigated design preferences and determinants of engagement with mental health apps by nondigitally native (middle-aged and older) adults who have chronic pain. Similar to findings across many other user populations, our participants in this population were generally avid mobile device users, and they strongly preferred to use apps that are free of charge, describe reassuring privacy policies, and add clear functional value to their lives [59]. Participants were also generally enthusiastic about health-related apps, particularly for tracking/logging purposes, and while on-demand access to app tools was clearly preferred, most participants also appreciated the potential value of occasional push notification “engagement reminders” if the notification timing was thoughtful and customizable [59]. Building on existing literature, this study also contributes some novel insights that are more unique to this nondigitally native population with chronic pain, when compared to younger users and users without chronic pain. Specifically, despite their daily use of mobile apps, many participants described frequent usability challenges, particularly related to app orientation and navigation (not just due to technical issues such as an app crashing). They were also more consistently motivated by “real-life” goal achievement and tangible health improvements than by gamification within an app, and many were wary of allowing apps to passively collect data, especially for the purpose of making inferences about their mental health. Furthermore, the majority of participants were unaware of apps designed to address chronic pain, but they showed a high level of interest in the concept. They were particularly interested in an infrastructure to track both their mood and pain symptoms and then identify associations between their symptoms, their activity on the app, and other life events. Finally, the participants cautioned against an overly cheerful, “infantile,” or informal tone for an app that is intended to support adult users with serious issues related to mental health and chronic pain.

### Comparison With Prior Work

The constellation of preferences that participants expressed presents a challenge when designing and delivering a health-related app that meets both their stated preferences and needs. Specifically, participants stated that they prefer not to be excessively bothered by push notifications. Yet, they also prefer to self-report their symptoms rather than have their symptoms inferred from passive data, and they describe low motivation as a common challenge in daily life—which is consistent with previous studies that have observed reduced app engagement among users with a high severity of depressive symptoms and fatigue (such as due to chronic pain) [59]. Of note, depression and fatigue are also barriers to engagement with nondigital health interventions [60].

Ultimately, we conclude that despite growing technological potential for passively collected data to infer changes in users’ mental and physical health accurately [61,62], feedback from our participants suggests that at least at this time, digital health interventions for middle-aged and older adults should not rely heavily upon passively collected data. Rather, careful iteration and customization of push notification frequency, timing, and content have consistently proven to be a key strategy for optimizing engagement with digital health interventions among people across the age spectrum [63]. For this population, effective notifications may include reminders about available tools that the user previously found helpful, previews about pain neuroscience principles that could meaningfully impact the user’s mindset, and/or insights into how app engagement could help address functional challenges previously reported by the user. Adaptive timing of notification delivery based on users’ demonstrated engagement patterns may also improve acceptance and engagement [64], and any use of passively collected data (beyond app engagement data) should likely be offered as an opt-in feature, rather than a default feature. Quantitative analysis of engagement patterns and symptom severity in response to varied push notification strategies is warranted.

Another challenge is that participants preferred apps that are both free of charge but are also not filled with obtrusive advertisements. This is consistent with preferences among younger age groups in which 67% of people report only using free apps and an additional 22% only use apps that cost $5 or less [65]. Third-party reimbursement of digital health interventions, either as stand-alone therapeutics or as part of a treatment bundle offered by a health system, could address these financial preferences and also add legitimacy to the intervention in the eyes of potential users.

Participants’ strong interest in health-related tracking capabilities poses a unique conundrum for chronic pain-related apps, as well. While tracking metrics such as step count and sleep quality are essentially universally encouraged by health care professionals, an app that frequently prompts a user to report their pain intensity could unintentionally increase pain rumination and subsequent discomfort and impairment [66-68]. This phenomenon is supported by prior studies among different target user populations, which have simultaneously reported high popularity of pain tracking features and mixed feedback regarding the helpfulness of these features [69,70]. Accordingly, we propose that a chronic pain-related app may consider enabling the user to systematically log pain *interference* (and potentially add free-text notes about pain severity), rather than prompting the user to frequently reflect on pain intensity.

To have the greatest impact specifically for people who are struggling with chronic pain, we also need to improve awareness about the potential and availability of digital health interventions to deliver evidence-based pain management techniques such as cognitive behavioral therapy and mindfulness exercises. Based on participants’ responses in this study, middle-aged and older adults may be most interested and accepting of such an intervention if it is introduced and recommended by a trusted member of their health care team. To most effectively increase awareness, these discussions could occur in primary care and orthopedic clinics, as well as mental health care settings [71]. Feasible, sustainable uptake of this workflow would likely be facilitated by use of established billing codes for behavioral health integration, digital mental health treatment, and/or remote therapeutic monitoring.

### Strengths

The broad potential applicability of our findings across multiple health domains is a strength of this study. Although the therapeutic purpose and content of various health-related apps may be somewhat distinct, common themes related to target users’ preferred engagement techniques and usability features exist. Some findings from this study can be used to inform optimal design of other digital mental health interventions and/or other health-related apps for nondigitally native middle-aged and older adults, not just related to chronic pain but potentially for other purposes such as medication monitoring, organ failure symptom monitoring (eg, for heart failure, kidney disease, etc), or encouragement to engage in social interaction or physical activity.

### Limitations

A limitation of this study is that the study population preferentially represents middle-aged, White females from the United States who use the internet at least multiple times daily. Therefore, we may not have fully captured themes that are uniquely relevant for older adults and for adults from other geographical or sociotechnical backgrounds. Nevertheless, even though the majority of participants reported daily internet use, we still identified usability challenges and specific opportunities to tailor digital health experiences for this group. Of note, our findings represent participants’ *stated* usability and engagement preferences; quantitative assessment of participants’ *actual* engagement with apps that are concordant versus discordant with their stated preferences was outside the scope of this study.

### Conclusions

Nondigitally native adults who are avid mobile device users still often encounter usability challenges and report design preferences that likely differ somewhat from those of younger users. To optimally meet the preferences and needs of this population, digital health interventions should emphasize tangible health improvements (more so than gamification) that can be achieved by engaging with the intervention, potentially using a multidomain tracking feature if appropriate. This population is generally available to receive real-time messages such as for just-in-time adaptive interventions, but the frequency and timing of any push notifications should still be thoughtful and customizable. This population remains wary of allowing passive data collection of their personal information. In conclusion, digital health interventions designed to address conditions that are more common with increasing age should account for these preferences.

## Data Availability

The dataset generated and analyzed during this study is available in the National Institute of Mental Health (NIMH) Data Archive (NDA) [72].

## Appendix

Multimedia Appendix 1 Interview guide.

## Abbreviations

BIT: Behavioral Intervention Technology
CFIR: Consolidated Framework for Implementation Research
COREQ: Consolidated Criteria for Reporting Qualitative Research
DDBT: Discover, Design/Build, and Test
GAD-7: 7-item Generalized Anxiety Disorder
IRB: institutional review board
PHQ-9: 9-item Patient Health Questionnaire
