# Supplementary material for: Design Preferences for Mental Health Apps Among Nondigitally Native Adults With Chronic Pain: Qualitative Analysis
Source: JMIR Mhealth Uhealth. 2026 Aug 11;14:e87358. doi: 10.2196/87358 (PMC13507721; doi:10.2196/87358)
Supplement: Supplementary file 1 [file mhealth_v14i1e87358_app1.docx]

**Design preferences for mental health apps among non-digitally native adults with chronic pain: A qualitative analysis**

**INTERVIEW GUIDE**

1. Individual: What is your experience and comfort level with smartphones and apps?
2. Inner Setting: When is your phone turned on and within arm’s reach during the day?
3. Outer Setting: What makes you *more/less* likely to use technology (or your phone) for something that you used to do a different way (e.g., text message vs phone call, take pictures, video chat, online banking, GPS, calendars, notes/lists)?
4. Implementation Process: If you’re trying to do something new with technology (or your phone) and get lost or run into trouble, how do you troubleshoot? (What is your preferred way of learning new technology?)
5. Adaptable periphery / BIT Instantiation Components: *If experienced with apps:*
   a. What makes an app easier for you to use?
   b. What makes you more likely to use an app?
   c. What makes an app reminder helpful versus annoying?
6. Individual: What is your experience using health-related apps? (food logs, step counters)
7. Intervention / BIT Instantiation Components – Elements, Characteristics: What would (or did) make you interested in using a mental health / chronic pain app? (purpose, features, tools, interface)
8. BIT Instantiation Components – Workflow: *When* would using a mental health / chronic pain app be most helpful to you?
   a. When might you want “reminders” from the app about resources and strategies to manage your wellness (e.g., time of day, or situation specific)?
   b. How does your real life and/or phone life look different when you are less busy and/or more lonely?

Blue text indicates mapping to CFIR domains and/or BIT model elements.
